# AI-Empowered Computational Examination of Chest Imaging for COVID-19 Treatment: A Review

**DOI:** 10.3389/frai.2021.612914

**Published:** 2021-07-21

**Authors:** Hanqiu Deng, Xingyu Li

**Affiliations:** ^1^ Department of Electrical and Computer Engineering, University of Alberta, Edmonton, AB, Canada; ^2^ School of Aerospace Engineering, Beijing Institute of Technology, Beijing, China

**Keywords:** chest imaging, image analysis, severity assessment, COVID-19, prognosis prediction, ROI segmentation, diagnostic model, machine learning

## Abstract

Since the first case of coronavirus disease 2019 (COVID-19) was discovered in December 2019, COVID-19 swiftly spread over the world. By the end of March 2021, more than 136 million patients have been infected. Since the second and third waves of the COVID-19 outbreak are in full swing, investigating effective and timely solutions for patients’ check-ups and treatment is important. Although the SARS-CoV-2 virus-specific reverse transcription polymerase chain reaction test is recommended for the diagnosis of COVID-19, the test results are prone to be false negative in the early course of COVID-19 infection. To enhance the screening efficiency and accessibility, chest images captured *via* X-ray or computed tomography (CT) provide valuable information when evaluating patients with suspected COVID-19 infection. With advanced artificial intelligence (AI) techniques, AI-driven models training with lung scans emerge as quick diagnostic and screening tools for detecting COVID-19 infection in patients. In this article, we provide a comprehensive review of state-of-the-art AI-empowered methods for computational examination of COVID-19 patients with lung scans. In this regard, we searched for papers and preprints on bioRxiv, medRxiv, and arXiv published for the period from January 1, 2020, to March 31, 2021, using the keywords of COVID, lung scans, and AI. After the quality screening, 96 studies are included in this review. The reviewed studies were grouped into three categories based on their target application scenarios: automatic detection of coronavirus disease, infection segmentation, and severity assessment and prognosis prediction. The latest AI solutions to process and analyze chest images for COVID-19 treatment and their advantages and limitations are presented. In addition to reviewing the rapidly developing techniques, we also summarize publicly accessible lung scan image sets. The article ends with discussions of the challenges in current research and potential directions in designing effective computational solutions to fight against the COVID-19 pandemic in the future.

## 1 Introduction

COVID-19, caused by severe acute respiratory syndrome coronavirus 2 (SARS-CoV-2), was noted to be infectious to humans in December 2019 in Wuhan, China. Afterward, it swiftly spread to most countries around the world. People infected with COVID-19 present with have fever, cough, difficulty in breathing, and other symptoms, while there are also asymptomatic infectious patients (Bai et al., 2020).

For COVID-19 diagnosis, on the one hand, the reverse transcription polymerase chain reaction (RT-PCR) is a specific and simple qualitative analysis method for the detection of COVID-19 (Tahamtan and Ardebili, 2020). Despite its high sensitivity and strong specificity, the RT-PCR test has several limitations. First, false-negative results for the SARS-CoV-2 test are very common in clinical diagnosis of COVID-19 due to various factors, e.g., an insufficient amount of virus in a sample (Xiao et al., 2020). Second, the RT-PCR test provides a yes/no answer without any indication of disease progression. On the other hand, clinical studies have discovered that most COVID-19 patients, even in the early course of infection or without showing any clinical symptoms, possess common features in their lung scans (Hao and Li, 2020; Long et al., 2020; Salehi et al., 2020; Wong et al., 2020; Zhou et al., 2020a). These patterns in lung images are believed to be a complement to the RT-PCR test and thus form an alternative important diagnostic tool for the detection of COVID-19. Particularly, among various non-invasive techniques to view and examine internal tissues and organs in chest, ultrasound (US) does not depict the differences between COVID-19 and other viral types of pneumonia well and magnetic resonance imaging (MRI) suffers from long scan times and high costs. Consequently, CT scans and chest X-ray (CXR) are the widely used techniques in lung scans for the clinical diagnosis of COVID-19 (Vernuccio et al., 2020; Dong et al., 2021). Currently, chest imaging has been used for preliminary/emergency screening, monitoring, and follow-up check-ups in COVID-19 treatment in China and Italy.

AI-empowered computational solutions have been successfully used in many medical imaging tasks. Particularly to combat COVID-19, computational imaging technologies include, but are not limited to, lung and infection region segmentation, chest image diagnosis, infection severity assessment, and prognosis estimation. Compared to physician’s examination, computational solutions are believed to be more consistent, efficient, and objective. In literature, early works on chest image examination for COVID-19 patients have usually adopted the paradigm of supervised learning to build an image analysis model. These learning algorithms range from support vector machine (SVM), K-nearest neighbor, random forest, decision tree to deep learning. Lately, to improve learning models’ generalization, transfer learning, multi-task learning, and weakly supervised learning have become popular.

In this article, we review the state-of-the-art AI diagnostic models particularly designed to examine lung scans for COVID-19 patients. To this end, we searched for papers and preprints on bioRxiv, medRxiv, and arXiv published for the period from January 1, 2020, to March 31, 2021, with keywords of COVID, lung scans, and AI. After quality inspection, 96 papers were included in this article, among which most are peer-reviewed and published in prestigious venues. We also included a small portion of reprints in this review due to their methodology innovations. Particularly, this review presents in-depth discussions on methodologies of region-of-interest (ROI) segmentation and chest image diagnosis in Segmentation of *Region of Interest in Lung Scans and COVID-19 Detection and Diagnosis*, respectively. Infection severity assessment and prognosis prediction from COVID-19 lung scans are closely related and thus presented together in *COVID-19 Severity Assessment and Prognosis Prediction*. Since AI solutions are usually data-driven, *Public COVID-19 Chest Scan Image Sets* lists primary COVID-19 lung image sets publicly accessible to researchers. Limitations and future directions on AI-empowered computational solutions to COVID-19 treatment are summarized at the end of this article.

Several review papers have been published on AI solutions to combat COVID-19. Pham et al. (2020) and Latif et al. (2020) have emphasized the importance of artificial intelligence and big data in responding to the COVID-19 outbreak and preventing the severe effects of the COVID-19 pandemic, but computational medical imaging was not their focus. (Dong et al. (2021) and Roberts et al. (2021) have broadly covered the use of various medical imaging modalities for COVID-19 treatment and Shi et al. (2021) overviewed all aspects along the chest imaging pipeline, from imaging data acquisition to image segmentation and diagnosis. This article constitutes the latest technical review (up to March 31, 2021) of AI-based lung scan screening for the COVID-19 examination. In contrast to previous review papers, this review particularly focuses on the AI-driven techniques for COVID-19 chest image analysis. We present an in-depth discussion on various AI-based methods, from their motivations to specific machine learning models and architectures. The specific scope and updated, in-depth review of technology distinguish this article from previous works.

## 2 Segmentation of Region of Interest in Lung Scans

The region of interest in lung scans is usually lung fields, lesions, or infection regions. As a prerequisite procedure, obtaining accurate segmentation of lung field or other ROIs in chest images is essential. It helps avoid the interference of non-lung regions in subsequent analysis (Majeed et al., 2020). This section provides a comprehensive review of AI-driven solutions for ROI segmentation for COVID-19 treatment. We will start with performance metrics for segmentation evaluation. Then, computational solutions are grouped based on image modalities (first with CXR, followed by CT). Note that though many studies have focused on lung segmentation, this article surveys publications directly related to COVID-19 treatment.

### 2.1 Performance Metrics

Dice coefficient is the most common metric used to evaluate segmentation methods. It quantifies the agreement between ground truth mask and segmentation results. Specifically, Dice coefficient is defined as follows:

$$Dice\left(X,Y\right)=\frac{2|A\cap B|}{\left|A|+|B\right|},$$ (1)

where *A* and *B* are ground truth and segmented regions, respectively; ∩ denotes the operation to obtain the overlap regions between *A* and *B*; |⋅| calculates the number of pixels in an image. In addition to Dice coefficient, accuracy, sensitivity, and precision can also be used as evaluation criteria (Yan et al., 2020).

### 2.2 Methodologies

Among various machine learning methods for ROI segmentation, the encoder-decoder architecture such as U-Net (Ronneberger et al., 2015) is the common backbone model. The encoder extracts numerical representations from a query image and the decoder generates a segmentation mask in the query image size. To boost the performance of U-Net shape models, different deep learning strategies are investigated to address unique challenges that exist in lung scans of COVID-19 patients. We specify these novel algorithms and models as follows.

#### 2.2.1 Region-of-Interest Segmentation in Chest X-Ray

Chest X-ray images from COVID-19 patients usually suffer from various levels of opacification. This opacification masks the lung fields in CXRs and makes accurate segmentation of lung fields difficult. To tackle this problem, Selvan et al. (2020) have proposed a weak supervision method that fuses a U-Net and a variational autoencoder (VAE) to segment lungs in high-opacity CXRs. The novelty in their method is the use of VAE for data imputation. In addition, three data augmentation techniques are attempted to improve the generalization of the proposed method.

#### 2.2.2 Region-of-Interest Segmentation in Computed Tomography Scans

In the literature, many studies have proposed improvements for ROI segmentation in lung CT images. For instance, an attention mechanism is often deployed for segmentation recently. For automated segmentation of multiple COVID-19 infection regions, Chen X. et al. (2020) have applied the soft attention mechanism to improve the capability of U-Net to detect a variety of symptoms of the COVID-19. The proposed aggregated residual transformation facilitates the generation of a robust and descriptive feature representation, further improving the segmentation performance. Inf-Net (Fan et al., 2020) is a semi-supervised segmentation framework based on a randomly selected propagation strategy. It utilizes implicit reverse attention and explicit edge attention to enhance abstract representations and model boundaries of lung infection regions, respectively. Similar to Inf-Net, COVID-SegNet proposed by Yan et al. (2020) introduces two attention layers in a novel feature variation (FV) block for lung infection segmentation. The channel attention handles confusing boundaries of COVID-19 infection regions, and the spatial attention in the FV block optimizes feature extraction in the encoder model.

Alternatively, multi-task learning is used to leverage useful information in multiple related tasks to boost the performance of both segmentation and classification (Amyar et al., 2020). In this study, a common encoder is shared by two decoders and one classification layer for COVID-19 infection segmentation, lung image reconstruction, and CT image binary classification (i.e., COVID-19 and non-COVID-19). Similarly, Wu et al. (2021) have designed a joint classification and segmentation framework, which used a decoder to map the combined features from the classification network and an encoder to the segmentation results.

In addition to the development of advanced deep learning models, several studies have tried to improve the segmentation performance by either synthesizing CT image samples or massaging image labels. For instance, Liu et al. (2020) have proposed using GAN to synthesize COVID-19 opacity on normal CT images. To address data scarcity, Zhou. et al. (2020) have created a CT scan simulator that expands the data by fitting variations in the patient’s chest images at different time points. Meanwhile, they have transformed the 3D model into three 2D segmentation tasks, thus not only reducing the model complexity but also improving the segmentation performance. On the other hand, instead of generating new, “fake” CT images for training, Laradji et al. (2020) have built an active learning model for image labeling. The active image labeling and infection region segmentation are iteratively performed until performance converges. Wang et al. (2020) have introduced a noise-robust Dice loss to improve the robustness of the model against noise labels. In addition, an adaptive self-ensembling framework based on the teacher-student architecture was incorporated to further improve noise-label robustness in image segmentation.

## 3 COVID-19 Detection and Diagnosis

Pneumonia detection from lung images is a key part of an AI-based diagnostic system for fast and accurate screening of COVID-19 patients. In this regard, machine learning methods, especially discriminative convolutional neural networks (CNN), are deployed for COVID-19 detection (binary classification of COVID-19 and non-COVID-19) and multi-category diagnosis (classification of normal, bacterial, COVID-19, and other types of viral pneumonia).

### 3.1 Performance Metrics

The widely used measurement metrics for image classification are accuracy, precision, sensitivity, specificity, and F1 score. The areas under the ROC curve (AUC) were also reported in some studies. ROC curve describes the performance of a classification model at various classification thresholds and AUC measures the area underneath the obtained ROC curve.

$$Accuracy=\frac{T_{P}+T_{N}}{T_{P}+T_{N}+F_{P}+F_{N}},$$ (2)

$$Precision=\frac{T_{P}}{T_{P}+F_{P}},$$ (3)

$$Sensitivity=\frac{T_{P}}{T_{P}+F_{N}},$$ (4)

$$Specificity=\frac{T_{N}}{T_{N}+F_{P}},$$ (5)

$$F1\;score=\frac{2T_{P}}{2T_{P}+F_{N}+F_{P}},$$ (6)

where $T_{P}$ is true positive, $T_{N}$ is true negative, $F_{P}$ is false positive, and $F_{N}$ is false negative.

### 3.2 Methodologies

The biggest challenge in the problem of COVID-19 detection from chest images is data scarcity. To address this issue, early works have usually designed diagnostic systems following the handcraft engineering paradigm. Moreover, solutions based on transfer learning, ensemble learning, multi-task learning, semi-supervised learning, and self-supervision have been proposed in recent publications. For ease of comparison, we summarize the reviewed methods for CXRs and CT scans in Table 1 and Table 2, respectively.

**TABLE 1:** Summary of COVID-19 detection from CXR images in the literature (%). If multiple models are used in a study, we report the best-performance model here.

Literature	Task	Method	Acc	Sens	Spec	Pre	F1	AUC
Wang et al. (2020a)	Multi-class	COVID-net	93.3	91.0	-	98.9	-	-
Asif et al. (2021)	Multi-class	InceptionV3	96.0	-	-	-	-	
Asnaoui and Chawki (2020)	Multi-class	InceptionResnetV2	92.18	92.11	96.06	92.38	92.07	-
Rajaraman and Antani (2020)	Two classes	VGG-16	93.08	97.11	86.49	92.16	94.57	95.65
Moutounet-Cartan (2020)	Multi-class	VGG-16	84.1	87.7	-	-	-	97.4
Albahli (2020)	Multi-class	Resnet	89	-	-	-	-	-
Eldeen et al. (2021)	Multi-class	Alexnet	87.1	-	-	91.67	89.53	-
Punn and Agarwal (2021)	Multi-class	NASNetLarge	95	90	92	95	90	94
Ozcan (2020)	Multi-class	Resnet50	97.69	97.26	97.90	95.95	96.60	-
Kumar et al. (2020)	Two classes	DeQueezeNet	94.52	96.15	-	90.48	-	-
Goodwin et al. (2020)	Two classes	Ensemble learning	89.4	80	-	53.3	64.0	-
Chatterjee et al. (2020)	Multi-class	Ensemble learning	88.9	85.1	-	-	86.9	-
Shibly et al. (2020)	Two classes	Faster R-CNN	97.36	97.65	95.48	99.29	98.46	-
Fakhfakh et al. (2020)	Two classes	ProgNet	92.4	93.9	-	93.0%	93.4	-
Li et al. (2020)	Multi-class	DCSL	97.01	97.09	-	97.00	96.98	-
Yamac et al. (2020)	Multi-class	CSEN	95.9	98.5	95.7	-	-	-
Al-karawi et al. (2020)	Multi-class	SVM	94.43	95.00	93.86	-	-	-
Khuzani et al. (2020)	Multi-class	CNN	94.05	100	-	96	98	-
Medhi et al. (2020)	Two classes	CNN	93	-	-	-	-	-
Abbas et al. (2021a)	Multi-class	4S-DT	97.54	97.88	97.15	-	-	99.58
Zhang et al. (2021)	Two classes	CAAD	72.77	71.7	73.83	-	-	83.61
Ahishali et al. (2021)	Two classes	CSEN	95.66	97.28	95.52	-	-	-
Zabirul-Islam et al. (2020)	Multi-class	LSTM	99.4	99.3	99.2	-	98.9	99.9
Narayan Das et al. (2020)	Multi-class	Xception	97.41	97.09	97.30	-	96.97	-
Khan et al. (2020)	Two classes	Xception	99.0	-	98.6	98.3	98.5	-
Abbas et al. (2021b)	Multi-class	VGG19	93.1	100	-	-	-	-
Apostolopoulos and Mpesiana (2020)	Multi-class	MobileNet V2	96.78	98.66	96.46	-	-	-
Minaee et al. (2020)	Multi-class	ResNet	-	98.0	90.7	86.9	-	98.9
Afshar et al. (2020)	Multi-class	CAPS	98.3	80	98.6	-	-	-
Toǧaҫar et al. (2020)	Multi-class	Ensemble learning	98.25	99.32	99.37	99.66	-	-
Apostolopoulos et al. (2020)	Multi-class	MobileNet V2	87.66	97.36	99.42	-	-	-

Acc, accuracy; AUC, area under the ROC curve; F1, F-score; Pre, precision; Sens, sensitivity and Spec, specificity.

**TABLE 2:** Summary of COVID-19 detection from lung CT slides in the literature (%). If multiple models are used in a study, we report the best-performance model here.

Literature	Task	Method	Acc	Sens	Spec	Pre	F1	AUC
Soares et al. (2020)	Two classes	xDNN	97.38	95.53	-	99.16	97.31	97.36
Anwar and Zakir (2020)	Multi-class	EfficientNetB4	89.7	-	-	-	89.6	89.5
Han et al. (2020)	Multi-class	AD3D-MIL	94.3	90.5	-	95.9	92.3	98.8
Rahimzadeh et al. (2020)	Two classes	ResNet50V2	98.49	94.96	98.7	81.26	-	-
He et al. (2020a)	Multi-class	DenseNet3D	87.62	89.07	91.13	85.94	87.48	89.8
Javaheri et al. (2021)	Multi-class	CovidCTNet	87.5	87.5	93.85	-	-	95
Di et al. (2021)	Two classes	UVHL	89.79	93.27	84.00	90.65	-	-
Chao et al. (2021)	Two classes	Random forest	88.4	84.3	-	-	-	88.0
He et al. (2020b)	Two classes	DenseNet-169	86	-	-	-	85	94
Jin et al. (2020)	Multi-class	ResNet152	-	90.19	95.76	-	-	97.17
Zhang et al. (2020)	Multi-class	Two-stage model	92.49	94.93	91.13	-	-	97.97
Aslan et al. (2021)	Multi-class	BiLSTM	98.70	-	99.33	98.77	98.76	99.00
Wang et al. (2021b)	Two classes	Inception	82.5	75.0	86.0	-	-	-
Song et al. (2021)	Two classes	DRE-net	86	96	-%	79	87	95

Acc, accuracy; AUC, area under the ROC curve; F1, F-score; Pre, precision; Sens, sensitivity and Spec, specificity.

#### 3.2.1 COVID-19 Detection/Diagnosis From Chest X-Ray

Handcrafted engineering is believed to be effective when prior knowledge of the problem is known. It is also preferred over deep learning when a training set is small. To solve the problem of COVID-19 diagnosis from chest X-ray images, Al-karawi et al. (2020) have proposed manually extracting image texture descriptors (LBP, Gabor features, and histograms of oriented gradient) for downstream SVM classification. Similarly, Khuzani et al. (2020) have suggested extracting numerical features from both spatial domain (Texture, GLDM, and GLCM) and frequency domain (FFT and Wavelet). Instead of SVM, a multi-layer neural network was designed for triple classification (normal, COVID-19, and other pneumonia). In the diagnostic pipeline introduced by Medhi et al. (2020), a CXR image is first converted to the grayscale version for image thresholding. Then, the obtained binary images are passed to a shallow net to distinguish normal images and COVID-19 cases. Chandra et al. (2021) have proposed extracting 8,196 radiomic texture features from lung X-ray images and differentiated different pneumonia types by multi-classifier voting.

Recently, with the increase in the collections of CXR images from COVID-19 patients, deep learning methods have become a major technique. Wang L. et al. (2020) have designed a lightweight diagnostic framework, namely, COVID-Net, for triple classification (i.e., no infection, non-COVID-19 infection, and COVID-19 viral infection). The tailored deep net makes heavy use of a residual projection-expansion-projection-extension (PEPX) design pattern and enhances representational capacity while maintaining relatively low computational complexity. Zabirul-Islam et al. (2020) have introduced an interesting hybrid deep CNN-LSTM network for COVID-19 detection. In this work, deep features of a CXR scan are extracted from a tailored CNN and passed to a long short-term memory (LSTM) unit for final classification. Since LSTM replaces a fully connected layer in the CNN-LSTM model, the number of trainable parameters in the model is reduced due to the parameter sharing property of LSTM.

Among the large volume of literature, transfer learning is one of the most common strategies in deep learning to combat data scarcity. It retrains a deep model on large-scale datasets and fine-tunes it on target COVID-19 image sets (Ahishali et al., 2021; Apostolopoulos et al., 2020; Apostolopoulos and Mpesiana, 2020; Asnaoui and Chawki, 2020; Khan et al., 2020; Moutounet-Cartan, 2020; Narayan Das et al., 2020; Ozcan, 2020; Ozturk et al., 2020; Punn and Agarwal, 2021; Abbas et al., 2021b; Asif et al., 2021; Eldeen et al., 2021). These models include, but are not limited to, Inception, ResNet, VGG-16, NASNet, and AlexNet. To further leverage the discriminative power of different models, ensemble learning is deployed, where multiple deep nets are used to vote for the final results. For example, DeQueezeNet, proposed by Kumar et al. (2020), ensembles DenseNet and SqueezeNet for classification. Similar models were proposed by Goodwin et al. (2020); Chatterjee et al. (2020); Shibly et al. (2020); Minaee et al. (2020); Toǧaҫar et al. (2020) Alternatively, Afshar et al. (2020) haveintroduced a capsule network-based model for CXR diagnosis, where transfer learning is exploited to boost the performance. To “open” the black box in a deep learning-based model, Brunese et al. (2020) have introduced an explainable detection system where transferred VGG-16 and class activation maps (CAM) (Zhou et al., 2016) were leveraged to detect and localize anomalous areas for COVID-19 diagnosis. Furthermore, Majeed et al. (2020) have performed a comparison study on pretrained CNN models and deployed CAM to visualize the most discriminating regions. Based on the experimental results, Majeed et al. (2020) have recommended performing ROI segmentation before diagnostic analysis for reliable results. The study by Hirano et al. (2020) focused on the vulnerability of deep nets against universal adversarial perturbation (UAP) with the application of detecting COVID-19 cases from chest X-ray images. The experimentation suggests that deep models are vulnerable to small UAPs and that adversary training is a necessity.

Since direct transfer across datasets from different domains may lead to poor performance, researchers have developed various strategies to mitigate the effects of domain difference on transfer performance. Li et al. (2020) have proposed a discriminative cost-sensitive learning (DCSL) model for a triple-category classification between normal, COVID-19, and other types of pneumonia. It uses a pre-trained VGG16 as the backbone net, where the first 13 layers are transferred and the two top dense layers are refined using an auxiliary conditional center loss to decrease the intra-class variations in representation learning. Convolution Support Estimation Network (CSEN) (Ahishali et al., 2021; Yamac et al., 2020) targets bridging the gap between model-based methods and deep learning approaches. It takes the numerical representations from pre-trained ChXNet as input and innovates a non-iterative mapping for sparse representation learning. In addition, Zhou et al. (2021) have considered the problem of COVID-19 CXR image classification in a semi-supervised domain adaptation setting and proposed a novel domain adaptation method, namely, semi-supervised open set domain adversarial network (SODA). It aligns data distributions in different domains through domain adversarial training (Ganin et al., 2016). To address highly imbalanced image sets, Zhang et al. (2021) have formulated the task of differentiating viral pneumonia in lung scans into a one-class classification-based anomaly detection problem and proposed a confidence-aware anomaly detection model (CAAD). CAAD consists of a shared feature extractor derived from a pre-trained EfficientNet, an anomaly detection module, and a confidence prediction module. A sample is detected as a COVID-19 case if it has a large anomaly score or a small confidence score.

Another strategy to tackle the data scarcity issue is data augmentation. For instance, offline augmentation strategies, such as adjusting noise, shear, and brightness, are adopted to solve the data imbalance problem by Ucar and Korkmaz (2020). To further address the shortage of COVID-19 CXR images, Albahli (2020) and Waheed et al. (2020) have proposed using GAN to synthesize CXR images directly. To leverage a large amount of unlabeled data in COVID-19 CXR detection, Rajaraman and Antani (2020) have introduced a semi-supervised model to generate pseudo-annotation for unlabeled images. Then, recognizing COVID-19 pneumonia opacities is achieved based on these “newly” labeled samples. Similarly, Abbas et al. (2021a) have introduced a self-supervision method to generate pseudo-labels. With abstract representations generated by the bottleneck layer of an autoencoder, unlabeled samples are clustered for downstream training.

#### 3.2.2 Detecting COVID-19 From Lung Computed Tomography Slides

Transfer learning is still the most common technique among the diverse methods to detect COVID-19 from lung CT images (Anwar and Zakir, 2020; He et al., 2020b; Chowhury et al., 2020; Soares et al., 2020; Wang S. et al., 2021). Particularly, previous studies (He et al., 2020a; Ardakani et al., 2020) have built a benchmark to evaluate state-of-the-art 2D and 3D CNN models (e.g., DenseNet and ResNet) for lung CT slides classification. It is worth mentioning that in the study of Wang S. et al. (2021), the model also performed re-detection on the results of the nucleic acid testing. According to this study, fine-tuned deep models can detect false-negative results. In addition, a lightweight 3D network optimized by neural architecture search was introduced for comparison in the proposed benchmark. To address the issue of large domain shift between source data and target data in transfer learning, He et al. (2020b) have proposed a self-supervised transfer learning approach called Self-Trans. By integrating contrastive self-supervision (Chen T. et al., 2020) in the transfer learning process to adjust the network weights pre-trained on source data, the bias incurred by source data is reduced in the target task. Aslan et al. (2021) have introduced a hybrid pre-trained CNN model and BiLSTM architecture to form a detection framework to improve the diagnosis performance.

In addition to transfer learning, diagnostic solutions based on weak supervision, multi-instance learning, and graphic learning were proposed in the literature. Rahimzadeh et al. (2020) have introduced a deep model that combined ResNet and the feature pyramid network (FPN) for CT image classification. ResNet is used as the backbone network and FPN generates a feature hierarchy from the backbone net’s features at different scales. The obtained feature hierarchy helps detect COVID-19 infection in different scales. DRE-Net proposed by Song et al. (2021) has a similar architecture that combines ResNet and FPN to achieve detail relation extract for image-level prediction. This study also implements Grad-CAM on ResNet layers for main lesion region visualization. Javaheri et al. (2021) have introduced a multi-step pipeline of a deep learning algorithm, namely, CovidCTNet, to detect COVID-19 from CT images. Using controlled CT slides as a reference, the dual function of BCDU-Net (Azad et al., 2019) in terms of anomaly detection and noise cancellation was exploited to differentiate COVID-19 and community-acquired pneumonia anomalies. An attention-based deep 3D multiple instance learning (AD3D-MIL) was proposed for accurate and interpretable screening of COVID-19 with weak labels (Han et al., 2020). In the AD3D-MIL model, a bag of raw CT slides is transformed to multiple deep 3D instances. Then, an attention-based pool layer is utilized to generate a Bernoulli-distributed bag label. COVID-19 and community-acquired pneumonia (CAP) have very similar clinical manifestations and imaging features in CT images. To differentiate the confusing cases in these two groups, Di et al. (2021) have designed an uncertainty vertex-weighted hypergraph learning (UVHL) method to identify COVID-19 from CAP. In this method, a hypergraph structure is constructed where each vertex corresponds to a sample and hyperedges connect neighbor vertices that share common features. Hypergraph learning is repeated till the hypergraph is converged.

Alternatively, instead of directly detecting COVID-19 from CT scans using one deep model, some researchers have proposed AI-based diagnosis systems that consist of multiple deep models, each completing one sub-task in sequential order. For example, Jin et al. (2020) have introduced an AI system that consisted of five key parts: 1) lung segmentation network, 2) slice diagnosis network, 3) COVID-infectious slice locating network, 4) visualization module for interpreting the attentional region of deep networks, and 5) image phenotype analysis module for explaining the features of the attentional region. By sequentially completing the key tasks, the whole system achieves 97.17% AUC on an internal large CT set. Zhang et al. (2020) have innovated a two-stage model to distinguish novel coronavirus pneumonia (NCP) from other types of pneumonia and normal controls in CT scans. Particularly, a seven-category lung-lesion segmentation model is deployed for ROI mask and the obtained lung-lesion map is fed to a deep model for COVID-19 diagnosis. Similarly, Wang B. et al. (2021) have introduced a diagnosis system consisting of a segmentation model and a classification model. The segmentation model detects ROI from lung scans and then the classification model determines if it is associated with COVID-19 for each lesion region.

## 4 COVID-19 Severity Assessment and Prognosis Prediction

Though most works on COVID-19 focus on ROI segmentation and chest image diagnosis, severity assessment and prognosis prediction are of significance. Severity assessment facilitates monitoring the COVID-19 infection course. Furthermore, it is closely related to prognosis outcomes (Fang et al., 2021), and detection of high-risk patients with early intervention is highly important to lower the fatality rate of COVID-19. Thus, we reviewed AI algorithms and models proposed for COVID-19 severity assessment and prognosis prediction in one section. Note that though it is closely related to severity assessment, prognosis prediction is a very difficult and challenging task. It requires monitoring patients’ outcomes over time, spanning from several days to several weeks. Given this challenge in data collection, the research on prognosis prediction relatively lags behind compared to COVID-19 detection and diagnosis.

### 4.1 COVID-19 Severity Assessment

#### 4.1.1 Performance Metrics

To evaluate the quality of COVID-19 severity estimation, we used Spearman’s rank correlation coefficient between the ground truth and prediction as the evaluation metric. Spearman’s *ρ* is defined as follows:

$$\rho \left(y^{true},y^{pred}\right)=\frac{cov\left(rg\left(y^{true}\right),rg\left(y^{pred}\right)\right)}{\sigma \left(rg\left(y^{true}\right)\right)\cdot \sigma \left(rg\left(y^{pred}\right)\right)},$$ (7)

where $y^{true}$ is the ground truth of infected fractions, $y^{pred}$ is the predicted fractions, cov (·,·) is a sample covariance, σ(⋅) is a sample standard deviation, and rg(⋅) is the rank vector of the input.

#### 4.1.2 Severity Assessment From Chest X-Ray

To assess the pneumonia severity in a CXR, Signoroni et al. (2020) have proposed a novel end-to-end scheme deploying U-Net++ as the backbone net. With the lung segmentation network (i.e., U-Net++), feature maps that come from different CNN layers of the encoder are masked with segmentation results and fed to a global average pooling layer with a SoftMax activation for final severity score. Cohen et al. (2020) have proposed a transfer learning-based method for assessing the severity of COVID-19 infection. With a pre-trained DenseNet as the backbone architecture, the convolutional layers transform an input image into a 1,024-dimensional vector and the dense layers serve as task prediction layers to detect 18 medical evidences for COVID-19 diagnosis. Finally, a linear regression model is deployed to fuse the 1024D features and 18 evidences for COVID-19 infection prediction.

#### 4.1.3 Severity Assessment From Computed Tomography Images

The severity of COVID-19 can be measured by different quantities. Goncharov et al. (2020) have proposed using infected lung percentage as an indicator of COVID-19 severity. In this regard, the study has deployed multi-task learning to detect COVID-19 samples and estimate the percentage of infected lung areas simultaneously. Since the method requires lung segmentation, U-Net is used as the backbone in the proposed multi-task learning. In the work proposed by Chao et al. (2021), an integrative analysis pipeline for accurate image-based outcome prediction was introduced. In the pipeline, patient metadata, including both imaging and non-imaging data, is passed to a random forest for outcome prediction. Besides, to address the challenges of weak annotation and insufficient data in COVID-19 severity assessment with CT, Li et al. (2021) have proposed a novel weak multi-instance learning framework for severity assessment, where instance-level augmentation was adopted to boost the performance.

### 4.2 COVID-19 Prognosis Prediction

Due to the complexity of prognosis estimation, previous studies usually fused lung ROI segmentation, COVID-19 diagnosis results, and patient’s metadata for a prognosis outcome. Note that in contrast to other tasks that follow similar evaluation protocols, AI-based prognosis prediction models are usually evaluated by different metrics in the literature. Depending on the specific setup and context, either classification accuracy or regression error can be used as model evaluation quantities. Thus, instead of summarizing the prognosis performance metrics in one sub-section independently, we will specify the evaluation protocols for each reviewed study in the following section.

#### 4.2.1 Prognosis Estimation From Chest X-Ray

To evaluate the COVID-19 course in patients for prognosis analysis, a deep model that leverages RNN and CNN architectures to assess the temporal evolution of images was proposed by Fakhfakh et al. (2020). The multi-temporal classification of X-ray images, together with clinical and radiological features, is considered as the foundation of prognosis and assesses COVID-19 infection evolution in terms of positive/negative evolution. Since this study formulates the prognosis prediction as a binary classification problem, conventional classification metrics, including accuracy, precision, recall, and F1 score, are reported.

#### 4.2.2 Prognosis Prediction From Computed Tomography Scans

Prior models of COVID-19 prognosis prediction from lung CT volume can be roughly categorized into two different scenarios. In the first scenario, prognosis prediction is formulated as a classification problem and the output is a classification result from a predefined outcome set (Meng et al., 2020; Chao et al., 2021; Shiri et al., 2021). For instance, Meng et al. (2020) have proposed a 3D DenseNet-similar prognosis model, namely, De-COVID19-Net, to predict a patient’s death. In this study, CT images are first segmented using a threshold-based method and the detected lung regions are fed into De-COVID19-Net. Before the final classification layer, clinic metadata and the obtained numerical features in De-COVID-Net are fused for the final prediction. Similarly, Shiri et al. (2021) have introduced an XGBoost classifier to predict patient’s survival based on radiomic features in lung CT scans and clinical data. Moreover, Chao et al. (2021) have implemented a prognosis model using a random forest to identify high-risk patients who need ICU treatment. Following a similar data processing flow from lung region segmentation, CT scan feature learning, metadata fusion to classification, a binary classification outcome in terms of ICU admission prediction is generated. For prognosis prediction models belonging to the first scenario, conventional classification evaluation metrics such as AUC and sensitivity are used.

In the second scenario, prognosis estimation is formulated by a regression problem (Wang S. et al., 2020; Zhang et al., 2020; Lee et al., 2021). Specifically, Zhang et al. (2020) have defined the prognosis output by the time in days that critical care demands are needed after hospital admission. In this regard, a light gradient boosting machine (LightGBM) and Cox proportional-hazards (CoxPH) regression model are built. The Kaplan–Meier analysis in model evaluation suggests that incorporating lung lesions and clinical metadata boosts prognosis prediction performance. Alternatively, Wang S. et al. (2020) have defined the prognostic event as the hospital stay time until discharge and proposed using two deep nets, one for lung region segmentation and the other for CT feature learning, for a multivariate Cox proportional hazard regression. In this study, Kaplan–Meier analysis and log-rank test are used to evaluate the performance of the proposed prognostic analysis. Under the same prognosis regression setting in (Wang S. et al., 2020), Lee et al. (2021) have developed a deep learning convolutional neural network, namely, Deep-COVID-DeteCT (DCD), for prognosis estimation based on the entire chest CT volume and experimentally demonstrates that multiple scans during hospitalization provide a better prognosis.

## 5 Public COVID-19 Chest Scan Image Sets

Machine learning is one of the core techniques in AI-driven computational solutions. Data are the stepstone to develop any machine learning-based diagnostic system. This section includes primary COVID-19 chest image sets that are publicly accessible to researchers. We will start with CXR datasets, followed by chest CT image sets. Note that when a dataset contains both X-ray images and CT scans, it will be summarized in the CXR section.

### 5.1 COVID-19 Chest X-Ray Datasets

COVID-19 CXR image data collection (Cohen et al., 2020) is an open public dataset of chest X-ray images collected from patients who are positive or suspected of COVID-19 or other types of viral and bacterial pneumonia (including MERS and SARS). The collection contains 589 chest X-ray images (542 frontal and 47 lateral views) from 282 people over 26 countries, among which 176 patients are male and 106 are female. Of the frontal views, 408 images are taken with standard frontal PA/AP (posteroanterior/anteroposterior) position and the other 134 are AP Supine (anteroposterior laying down). In addition to CXR, the dataset also provides clinical attributes, including survival, ICU stay, intubation events, blood tests, and location, and is free from clinical notes for each image/case.

BIMCV COVID-19 + (Vayá et al., 2020) is a large dat set with 1,380 chest X-ray images and 163 full-resolution CT scans from 1,311 patients in the Valencian Region Medical Image Bank (BIMCV). All samples are labeled as COVID-19 infection, no infection, and other infection and stored as 16-bit PNG format images. Along with chest images, metadata including radiographic findings, pathologies, polymerase chain reaction, IGG and IGM diagnostic antibody tests, and radiographic reports are also provided. In addition, ten images in this dataset are annotated by a team of eight radiologists from the Hospital Universitario de San Juan de Alicante to include semantic segmentation of radiographic findings.

COVID-19 Radiography Database (Chowhury et al., 2020) consists of 219 COVID-19 positive CXR images, 1,341 normal images, and 1,345 viral pneumonia images. All images are stored in grayscale PNG format with a resolution of 1024 by 1024 pixels.

### 5.2 COVID-19 Computed Tomography Scan Sets

COVID-CT-dataset (Yang et al., 2020) provides 349 CT scans with clinical characteristics of COVID-19 from 216 patients and 463 non-COVID-19 CTs. Images in this set are collected from COVID19-related papers from medRxiv, bioRxiv, NEJM, JAMA, and Lancet and thus in different sizes. The number of CT scans that a patient has ranges from 1 to 16, with an average of 1.6 per patient. The utility of these samples is confirmed by a senior radiologist who has been diagnosing and treating COVID-19 patients since the outbreak of the COVID-19 pandemic. Meta-information, including patient ID, patient information, DOI, and image caption, is available in this dataset.

COVID-CTset (Rahimzadeh et al., 2020) is a large CT images dataset that collected 15,589 COVID-19 images from 95 patients and 48,260 normal images from 282 persons from the Negin Medical Center located at Sari in Iran. The patient’s private information is removed and each image is stored in 16-bit grayscale TIFF format with 512*512-pixel resolution.

MosMedData (Morozov et al., 2020) contains 1,100 lung CT scans from municipal hospitals in Moscow, Russia, between March 1, 2020, and April 25, 2020. Among the 1,100 images, 42% are of male and 56% of female, with the rest 2% unknown. The dataset groups samples into five categories (i.e., zero, mild, moderate, severe, and critical) based on the severity of lung tissue abnormalities related to COVID-19, where the sample ratios of the five categories are 22.8, 61.6, 11.3,4.1, and 0.2%, respectively. In addition to severity labels, a small subset with 50 cases in MosMedData is annotated with binary ROI masks in the pixel level, which localizes the ground-class opacifications and regions of consolidations in CT images.

CC-CCII CT image set (Zhang et al., 2020) consists of a total of 617,775 CT images from 4,154 patients in China to differentiate between NCP due to SARS-CoV-2 virus infection, common pneumonia incurred by viral, bacterial, or mycoplasma, and normal controls. Each image is accompanied by corresponding metadata (patient ID, scan ID, age, sex, critical illness, liver function, lung function, and time of progression). Furthermore, 750 CT slices from 150 COVID-19 patients are manually annotated at the pixel level and classified into four classes: background, lung field, ground-glass opacity, and consolidation.

COVID-19 CT segmentation dataset (Jenssen, 2020) consists of 100 axial CT images associated with confirmed COVID-19 cases from the Italian Society of Medical and Interventional Radiology. Each image is segmented by a radiologist using three labels: ground-glass (mask value =1), consolidation (=2), and pleural effusion (=3) and stored in a single NIFTI file with a size of 512×512×110.

SARS-CoV-2 CT scan dataset (Soares et al., 2020) contains 1252 CT scans that are positive for SARS-CoV-2 infection and 1,230 images from non-COVID-19 patients from hospitals in Sao Paulo, Brazil. This dataset is used to develop artificial intelligence methods to identify if a person is infected by SARS-CoV-2 through the analysis of his/her CT scans.

## 6 Summary and Discussions on Future Works

AI and machine learning have been applied in the fight against the COVID-19 pandemic. In this article, we reviewed the state-of-the-art solutions to lung scan examination for COVID-19 treatment. Though promising results have been reported, many challenges still exist that should be discussed and investigated in the future.

First, when studying these publications, we find it very challenging to compare their performance. Prior works have usually evaluated model performance on either their private dataset or a combination of several public image sets. Furthermore, the use of different evaluation protocols (e.g., binary classification vs. multi-category classification) and various performance metrics makes the comparison very difficult. We argue that the lack of benchmark hinders the development of AI solutions based on state of the art. With more chest images being available, we expect a comprehensive benchmark for fair comparison among different solutions in the near future.

Second, AI-based methods, especially deep learning, usually require a huge amount of training data with quality annotations. It is always more difficult and expensive to collect medical images to collect natural image samples. Compared to the model sizes, which are easily up to millions of training parameters, the sample size in the current public lung scan image sets is relatively small. This observation is more noticeable in the literature of prognosis estimation. Consequently, the generalizability of the state-of-the-art models on unseen data is in question. In addition, since current lung scan image sets contain many images from heavily or critically ill patients, there is a debate on if AI can differentiate nuances between mild/moderate COVID-19 and other lower respiratory illnesses in real clinical settings. The data bias in training data would greatly harm model’s generalizability. Without tackling these data bias issues, data-driven solutions are hardly ready for deployment clinically. There are two possible solutions to address this issue. On the one hand, collecting large image sets that cover a variety of COVID-19 cases is demanding. On the other hand, methods based on self-supervision anomaly detection can help mitigate data bias in data-driven solutions. Specifically, it is relatively easier to collect a large number of lung scans from healthy subjects. By studying the normal patterns in these negative cases, AI-based anomaly detection methods are expected to detect positive chest images by identifying any abnormal patterns that do not follow the normal patterns.

Third, in COVID-19 treatment, examination based on data from one modality is usually not sufficient. For instance, some COVID-19 patients do not experience fever and cough, while others have no symptoms in their chest images. To tackle this problem, omni-modality learning capable of holistically analyzing patients’ clinical information, for example, blood test results, age, chest images, and RT-PCR test, is highly desired for COVID-19 treatment. We have witnessed the trend of including multi-modality data in prognosis estimation. However, from the technical aspect, current multi-modality data fusion methods are too simple. How to effectively combine the lung scans with patients’ clinical records is still an open question.

Last but not least, despite the promising results reported in prior arts, the issue of explainability in these AI models is less addressed. Decision-making in a medical setting can have serious health consequences; it is often not enough to have a good decision-making or risk-prediction system in the statistical sense. Conventional medical diagnosis and prognosis usually are concluded with evidence. However, such evidence is usually missed in current AI-based methods. We argue that this limitation of explainability is another hurdle in deploying AI technology on lung scans for COVID-19 examination. A desirable system should not only indicate the existence of COVID-19 (with yes/no) but also be able to identify what structures/regions in images are the basis for its decision.
